# Expression of S100A6 in Rat Hippocampus after Traumatic Brain Injury Due to Lateral Head Acceleration

**DOI:** 10.3390/ijms15046378

**Published:** 2014-04-15

**Authors:** Bo Fang, Ming Liang, Guitao Yang, Yuqin Ye, Hongyu Xu, Xiaosheng He, Jason H. Huang

**Affiliations:** 1 Department of Neurosurgery, Xijing Hospital, Fourth Military Medical University, Xi’an 710032, China; E-Mails: happyfangbo@gmail.com (B.F.); liangming777@163.com (M.L.); chinayeyuqin@163.com (Y.Y.); xuhy@fmmu.edu.cn (H.X.); 2 Department of Medical, Teaching and Research Administration, Xijing Hospital, Fourth Military Medical University, Xi’an 710032, China; E-Mail: guitaoy@hotmail.com; 3 Department of Neurosurgery, University of Rochester, Rochester, NY 14642, USA; E-Mail: jason_huang@urmc.rochester.edu

**Keywords:** S100A6, hippocampus, cognitive deficits, traumatic brain injury (TBI), rats

## Abstract

In a rat model of traumatic brain injury (TBI), we investigated changes in cognitive function and S100A6 expression in the hippocampus. TBI-associated changes in this protein have not previously been reported. Rat S100A6 was studied via immunohistochemical staining, Western blot, and reverse transcription-polymerase chain reaction (RT-PCR) after either lateral head acceleration or sham. Reduced levels of S100A6 protein and mRNA were observed 1 h after TBI, followed by gradual increases over 6, 12, 24, and 72 h, and then a return to sham level at 14 day. Morris water maze (MWM) test was used to evaluate animal spatial cognition. TBI- and sham-rats showed an apparent learning curve, expressed as escape latency. Although TBI-rats displayed a relatively poorer cognitive ability than sham-rats, the disparity was not significant early post-injury. Marked cognitive deficits in TBI-rats were observed at 72 h post-injury compared with sham animals. TBI-rats showed decreased times in platform crossing in the daily MWM test; the performance at 72 h post-injury was the worst. In conclusion, a reduction in S100A6 may be one of the early events that lead to secondary cognitive decline after TBI, and its subsequent elevation is tightly linked with cognitive improvement. S100A6 may play important roles in neuronal degeneration and regeneration in TBI.

## 1. Introduction

Traumatic brain injury (TBI) is very common in modern society and is associated with extremely high disability and death rates [1]. Limited insight into the molecular mechanisms after TBI has hindered the progress of developing treatments. It has been established that independent and collaborative functioning of hippocampus, amygdala, and dorsal striatum are responsible for the learning and memory deficits often observed in patients with mild or moderate TBI [2–4].

Many studies on the malfunction and degeneration of neurons following TBI have revealed that intracellular accumulation of calcium plays an important role in neuronal edema, apoptosis, repair, and regeneration [5]. The latest research has suggested that calcium overload is a common and last path to neuronal damage. Although the underlying mechanism involved in the intracellular transduction of the calcium signal is still unclear, S100 and its target are considered key molecules in the pathway [6].

S100 is widely expressed in the cell membranes, cytoplasm and nuclear membranes of various tissues. It is not known whether S100 has functional homogeneity across various tissues. An S100 protein family member called S100B was observed to be closely associated with TBI in experimental models and human patients and is even regarded as a biomarker of brain damage [7,8]. S100A6 (calcyclin binding protein, CacyBP) is another member of the S100 family and contains two EF-hand calcium-binding sites and has a low molecular weight. This protein is highly expressed in brain tissue and has been implicated in calcium homeostasis, regulation of cell motility, transport of other ions [9], ubiquitinylated proteolytic degradation [10], and neuronal proliferation [11]. S100A6 is an important component of a novel ubiquitinylation pathway regulating β-catenin degradation. It forms a complex with CacyBP/SIP(Siah-1 interacting protein) through its *C*-terminal fragment. Co-immunoprecipitation of this complex with S100B from brain cells and with S100A6 from Ehrlich ascites tumor cells suggests that these interactions are physiologically relevant and that the ubiquitinylation complex involving CacyBP/SIP might be regulated by S100 proteins [11]. Although structurally very similar, S100B and S100A6 interacted with different receptor for advanced glycation end products (RAGE) extracellular domains. These two proteins differentially modulated cell survival. At a micromolar concentration, S100B increased cellular proliferation, whereas at the same concentration, S100A6 triggered apoptosis. S100B recruited phosphatidylinositol 3-kinase/AKT and NF-κB, whereas S100A6 activated JNK (c-Jun *N*-terminal kinases), although both induced the formation of reactive oxygen species. Furthermore, S100B and S100A6 modulated cell survival in a RAGE-dependent manner; S100B specifically interacted with the RAGE V and C1 domains and S100A6 specifically interacted with the C1 and C2 RAGE domains [12].

S100A6 expression was observed in the hippocampus of mouse models of brain aging and epilepsy, and in the axotomized hypoglossal nucleus of mice [13]. However, there have been no reports of studies of S100A6 in TBI models or patients who had TBI. Because the hippocampus is important for animal cognition and is vulnerable to traumatic injury, this study, emphases on apoptosis post-TBI and has employs a rat model of TBI to study the expression of S100A6 in the hippocampus. Additionally, cognitive performance is assessed by the Morris water maze (MWM) test in order to better understand the functional effects of changes in S100A6 levels. We hypothesize that S100A6 plays an important biochemical and functional role in the process of neuronal degeneration and regeneration in the hippocampus following TBI via calcium signal transduction.

## 2. Results

### 2.1. Expression of Hippocampal S100A6: Immunohistochemical Staining, Protein, and mRNA Levels

**Figure 1.**
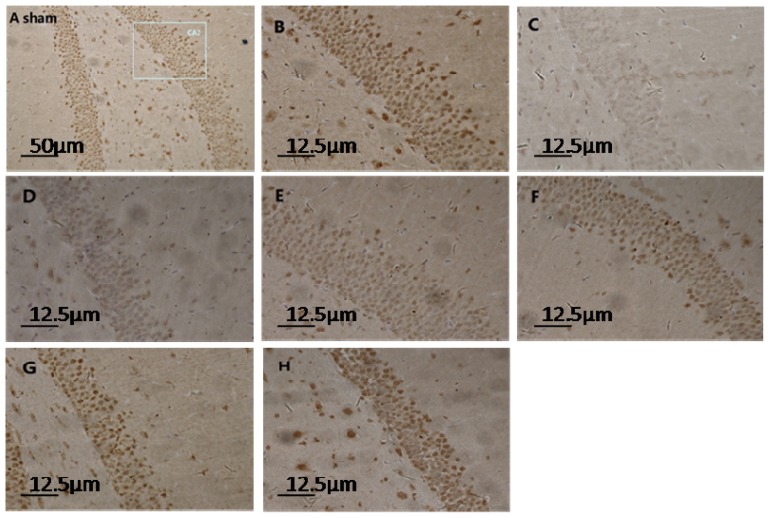
Light micrograph of hippocampus sections in sham- and TBI-rats at different time-points post-injury (immunohistochemistry for S100A6). The normal structure of the hippocampus from a sham rat is depicted in (**A**) (10 × 10); a rectangular section of the CA2 region is enlarged in (**B**) (40 × 10); Normal pyramidal neurons in sham rats were interspersed with condensed dark-brown neurons. In TBI-rats, the number and density of pyramidal neurons noticeably decreased at 1 h post-injury as shown in (**C**) (40 × 10); and began to increase gradually from 6 h to 12 h, 24 h, 72 h, and 14 day, as shown respectively in (**D**–**H**) (40 × 10). These neurons, which are full of densely-compacted fine particles, provide strong evidence of higher cellular S100A6 immunoreactivity.

**Figure 2.**
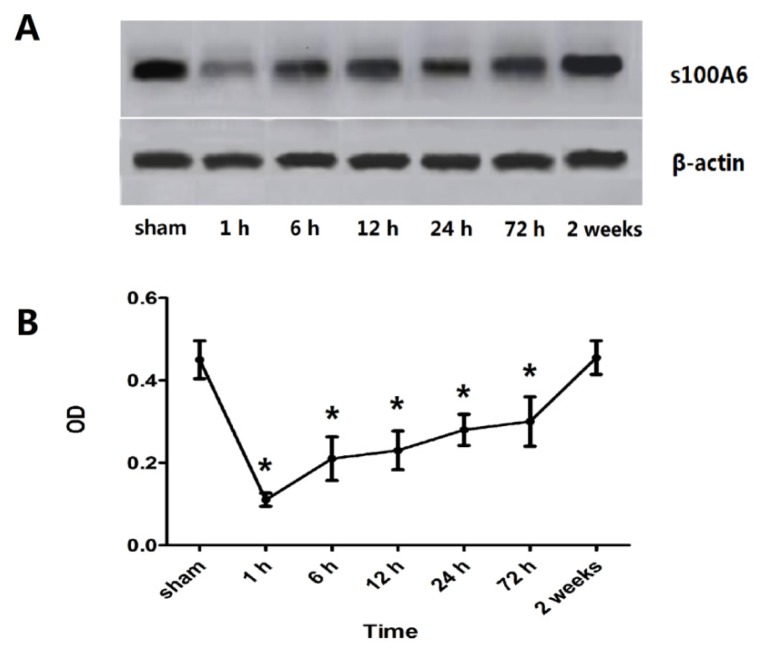
Expression of hippocampal S100A6 protein for sham- and TBI-rats. (**A**) A representative Western blot of S100A6 and β-actin. An early down-regulation of hippocampal S100A6 protein after TBI was observed, followed by increasing protein levels from 6 h post-TBI; (**B**) OD (optical density) values of S100A6 protein expression. The OD values mirrored the expression pattern of S100A6 observed by Western blot. This value was 0.45 ± 0.046, 0.11 ± 0.016, 0.21 ± 0.053, 0.23 ± 0.047, 0.28 ± 0.038, 0.30 ± 0.06, and 0.46 ± 0.041, respectively for the group of sham, 1 h, 6 h, 12 h, 24 h, 72 h, 2 weeks. An immediate down-regulation was seen at 1 h post-injury, followed by increasing expression from 6 h until 14 days when the level was akin to sham protein levels. A significant difference in OD values of TBI rats compared to sham rats was noted at the 1 to 72 h post-injury time points (* *p* < 0.05). Data are presented as mean ± SEM, *n* = 10.

**Figure 3.**
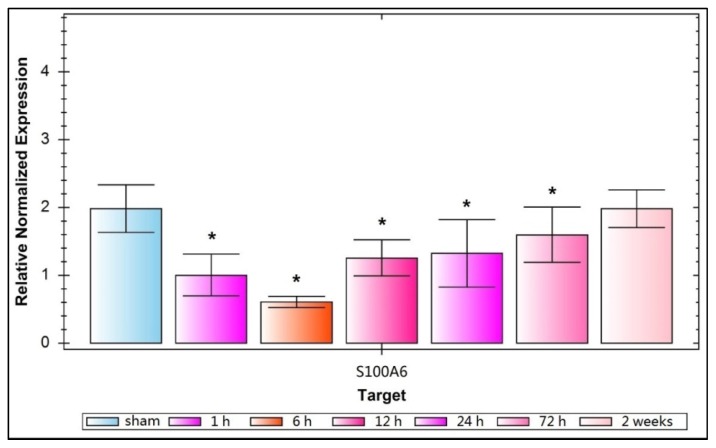
Relative normalized expression of S100A6 mRNA for sham- and TBI-rats. This relative normalized expression reflects that S100A6 mRNA is down regulated greatly 1 h after trauma, reaches its lowest point at 6 h, then increases gradually over 12, 24, and 72 h, and approaches sham levels at 14 days. This value is 1.983 ± 0.3513, 1.000 ± 0.3124, 0.6022 ± 0.0822, 1.245 ± 0.2713, 1.323 ± 0.4980, 1.591 ± 0.4121, and 1.981 ± 0.2778, respectively, for the group of sham, 1 h, 6 h, 12 h, 24 h, 72 h, 2 weeks. A significant decline of relative normalized expression of S100A6 mRNA occurrs in TBI-rats compared with sham-rats from 1 to 72 h post-injury (* *p* < 0.05). Data are presented as mean ± SEM, *n* = 10.

Microscope visualization showed that layers of pyramidal neurons were located in the CA2 region of the hippocampus of sham-rats (Figure 1A,B). Dark-brown fine particles were seen within neuronal cells. The density of these particles revealed the strength of S100A6 immunoreactivity. From 6 h post-TBI, an increase in density was observed with longer survival (Figure 1D–H). The S100A6 protein (Figure 2A,B) and S100A6 mRNA expression (Figure 3) in TBI rats showed a similar pattern across survival time-points.

### 2.2. Learning and Memory Dysfunction after TBI

**Figure 4.**
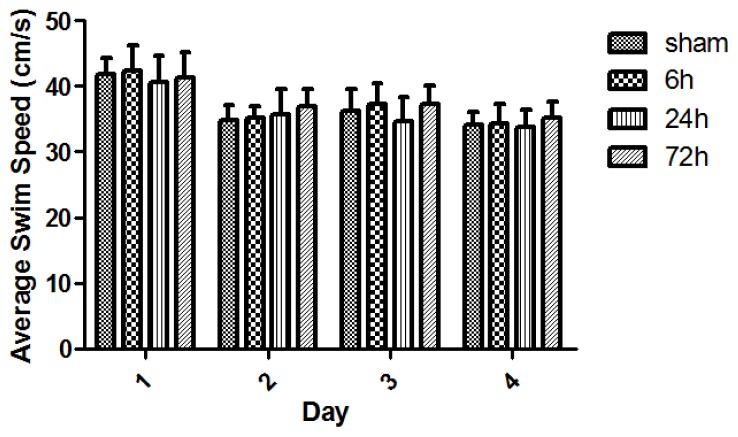
Swim speed (s) of sham- and TBI-rats with various survivals in daily Morris water maze (MWM) test. The animal swim-speed did not significantly differ between sham and TBI subgroups in daily MWM test (*p* > 0.05). Day1: The swim speed was 41.87 ± 2.49, 42.45 ± 3.87, 40.63 ± 4.11, and 41.31 ± 3.93 respectively for the group of sham, 6, 24, and 72 h. Day2: The speed was 34.81 ± 2.33, 35.10 ± 1.80, 35.78 ± 3.72, and 37.00 ± 2.63, respectively, for these above groups. Day3: The speed was 36.26 ± 3.31, 37.28 ± 3.12, 34.73 ± 3.61, and 37.37 ± 2.75, respectively, for these groups. Day4: The speed was 34.15 ± 1.82, 34.29 ± 3.00, 33.82 ± 2.63, and 35.25 ± 2.31, respectively, for these groups. Although nearly all subgroups of sham- and TBI-rats showed slightly faster speeds across the 4-day swimming trial, the speed differences were not statistically significant (*p* > 0.05). Data are presented as mean ± SEM, *n* = 8.

**Figure 5.**
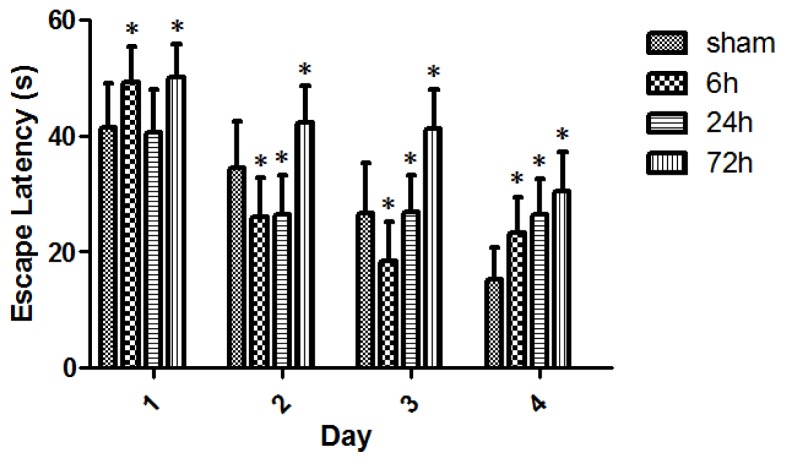
Escape latency (cm/s) for sham- and TBI-rats in daily MWM test. All sham- and TBI-subgroups had gradually-decreased escape latencies over the consecutive 4 days MWM test, indicating a learning curve. Day1: The escape latency was 41.50 ± 7.55, 49.20 ± 6.13, 40.62 ± 7.52, and 49.81 ± 6.63, respectively, for the group of sham, 6, 24, and 72 h. Day2: The escape latency was 34.44 ± 8.14, 25.75 ± 6.93, 26.29 ± 6.69, and 42.25 ± 6.26, respectively, for these above groups. Day3: The escape latency was 26.72 ± 8.59, 18.45 ± 6.31, 26.89 ± 6.30, and 41.24 ± 6.73, respectively, for these groups. Day4: The escape latency was 15.32 ± 5.36, 23.20 ± 6.11, 26.53 ± 5.92, and 30.36 ± 6.83, respectively, for these groups. With each MWM test day, 72 h survived-rats demonstrated a significant increase in escape latency. However, TBI-rats did not have an expected and stable delay in escape latency across 6 and 24 h survival on the 1st, 2nd, and 3rd day’s MWM tests. On the 4th day of MWM test, an apparent gradual delay in escape latency across 6, 24, and 72 h was observed in TBI-rats. Data are presented as mean ± SEM, *n* = 8. * *p* < 0.05, compared with sham-rats.

**Figure 6.**
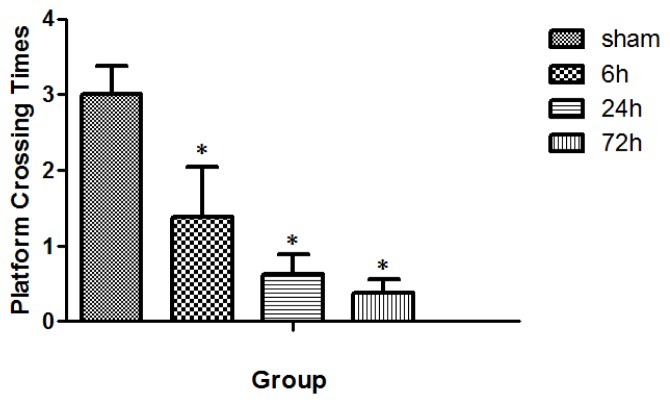
Times of platform crossing in sham- and TBI-rats 3 days after the 4-day MWM trial. The value was 3.00 ± 0.38, 1.38 ± 0.67, 0.63 ± 0.26, and 0.38 ± 0.18, respectively, for the group of sham, 6, 24, and 72 h. Three days after the 4-days MWM trial, TBI-rats spent significantly less time crossing the platform than the sham rats, with larger differences observed for rats with longer survival (* *p* < 0.05). Data are presented as mean ± SEM, *n* = 8.

All rats swam and found the platform. The animal swim speeds in daily MWM trials were not significantly different between sham and TBI subgroups (*p* > 0.05) (Figure 4). The observed escape latency for sham- and TBI-animals in MWM test over four consecutive days is shown in Figure 5. Three days after finishing the 4-day MWM trial, a probe test showed that TBI subgroups with various survivals spent less times crossing the platform compared with the sham group (Figure 6).

## 3. Discussion

The present study demonstrated that TBI-rats showed a lower expression of hippocampal S100A6 early post-injury, accompanied by relatively delayed deficiencies in spatial learning and memory. The down-regulation of S100A6 protein expression occurred as early as 1 h after TBI, followed by a gradual up-regulation across 6, 12, 24, and 72 h, and finally reaching sham-levels at 14 days (Figure 2). The mRNA expression of S100A6 was also low at 1 h, and maintained at a low level at 6 h. Afterwards, mRNA levels increased until they recovered to sham levels at 14 days (Figure 3). A similar pattern in both S100A6 protein and mRNA expression levels was observed from posttraumatic 1 h to 14 days.

A recent study reported for the first time that up-regulation of S100A6 in activated astrocytes may be linked to glutamate toxicity in the hippocampus of two mouse models of brain aging and epilepsy, and in the axotomized hypoglossal nucleus of one mouse model [13]. The authors noted that no causal conclusions could be drawn based on their correlative findings. Despite this weakness, the authors suggested that their data provided some insights into the association between astrocytic S100A6 and glutamate-induced neuronal damage under pathophysiological conditions [13].

However, the results of the present study were not similar to the above reports. We found that there was an immediate and significant reduction in S100A6 protein and mRNA expression 1–6 h post trauma, followed by a gradually-increased expression of S100A6 across 12 h, 24 h, 72 h, and 14 days. Perhaps the increased protein expression provided biochemical substrates required for neuronal regeneration and repair. The disparity between the current study and the previous report in the literature could derive from differences in experimental models and the time-points tested.

Calcium-dependent phosphorylation of CacyBP/SIP is initiated and driven by its interactions with S100A6 [14]. The present study suggests that the level of S100A6 available to form a complex is low immediately post-TBI. Other studies have shown that immediate calcium influx into neuronal cells leads to a cascade of secondary events, including a phosphorylation of CacyBP/SIP at the expense of prompt down-regulation of S100A6 [15,16]. Analysis of CacyBP/SIP mRNA expression during rat brain development showed its highest level in the cerebellum at postnatal Day 21, which might suggest the involvement of CacyBP/SIP in the development of rat brain [17]. It demonstrated, although not directly, that S100A6 would be highly expressed in the neuronal growth of the brain [18]. TBI- and sham-rats all revealed an apparent learning curve expressed in escape latency (Figure 5). In spite of the fact that TBI-rats had relatively poorer spatial cognitive abilities than the sham-rats, the disparity between them was not significant early post-injury. There were marked cognitive impairments in TBI-rats with a survival as long as 72 h at each daily MWM test compared with sham animals. In the probe test of MWM, TBI-rats crossed the platform less times, and the animals that survived 72 h performed the worst.

The posttraumatic expression of S100A6 increased in TBI-rats at 72 h post-injury, even though the rats exhibited a much worse performance in MWM test. It is possible that the S100A6 protein changed dramatically prior to the factors that later influenced the animal’s spatial cognition. It should be noted that there were greater standard errors in escape latency (Figure 5), perhaps because of the independent group-between design used for the study. The escape latency in daily MWM test was longer in TBI-rats compared with sham-rats, although this tendency was the most stable and obvious on the 4th test day. Moreover, a gradually-decreased change in escape latency across four daily MWM tests was noted for each independent group of sham and TBI rats with different survivals, indicating a similar learning curve.

It is known that corticosteroids from the adrenal cortex, especially glucocorticoids, influence a variety of behaviors such as learning and memory and other cognitive activities. Glucocorticoid receptors (GRs) display higher concentrations in the area of the hippocampus, a brain region involved mainly in the regulation of spatial orientation and learning [19]. In our previous study, immunohistochemical staining, Western blot, and RT-PCR results indicated that a down-regulation of GRs expression occurred in the hippocampus among TBI-rats at 4, 5, 6, and 7 days post-injury. An independent subgroup of TBI-rats at 4 days post-injury demonstrated a worsened performance of learning and memory in MWM test. The similarly-timed pattern seems to suggest that reduced expression of hippocampal GRs is closely associated with cognitive deficits in TBI [20]. In reference to the relation to cognitive function, S100A6 changed at an earlier period post-injury than GRs, which had a much closer association with animal cognitive function.

It is known that CacyBP/SIP participates in further signal transduction after complexing with S100A6 in a calcium-dependent way [11,21]. This protein complex influences by ubiquitination, the degradation of β-catenin protein, which serves as a key moderator of neuronal survival [22–24]. Furthermore, S100A6/CacyBP/SIP directly combines with microtubulin and then constructs microtubules to form neuronal skeletons [25,26].

Hence, considering the occurrence of intracellular calcium overload immediately after brain injury trauma, it is possible that S100A6 transduces the calcium overload signal to CacyBP/SIP and then initiates the degradation of β-catenin, which reduces the stability of microtubules and leads to neuronal death.

## 4. Materials and Methods

### 4.1. Animals

Healthy adult male Sprague-Dawley rats (provided by the Center of Experimental Animals of the Fourth Military Medical University, Xi’an, China) weighing 200 ± 20 g were maintained in an environment with constant temperature, humidity, and a 12 h light/dark cycle (light on from 07:00 to 19:00). The animals had free access to food and water. All measures were taken to minimize animal pain or discomfort. All experimental procedures were performed in accordance with the National Experimental Animals Guidelines and approved by the Chinese Small Animal Protection Association.

### 4.2. TBI Model and Grouping of Animals

The study design was an independent between-group study. Rats were divided into a TBI group (*n* = 84) and a sham group (*n* = 32). Three subgroups of TBI-rats (eight in each subgroup) at 6, 24, and 72 h post-injury underwent four trials per day of Morris water maze (MWM) test for four consecutive days. Eight sham-rats also underwent the same MWM trials to provide a baseline performance. The rest of the TBI-rats (*n* = 60) were divided equally into six subgroups: Surviving for 1 h, 6 h, 12 h, 24 h, 72 h, or 14 days post-TBI, at which time the rats were sacrificed and prepared for immunohistochemical staining, Western blot and reverse transcription polymerase chain reaction (RT-PCR) to probe for S100A6. At each survival point, half of each rat brain (cut sagittally) was used for immunohistochemical staining (*n* = 10), and the other half was used for Western blot and then RT-PCR (*n* = 10). Right and left halves were counterbalanced across immunohistochemistry and Western blot/RT-PCR. The rest of the sham-rats (*n* = 24) served as controls. In order to establish baseline results, eight rats (16 halves of brains) were sacrificed for immunohistochemistry and 16 rats (32 halves of brains) were sacrificed for Western blot/RT-PCR.

**Figure 7.**
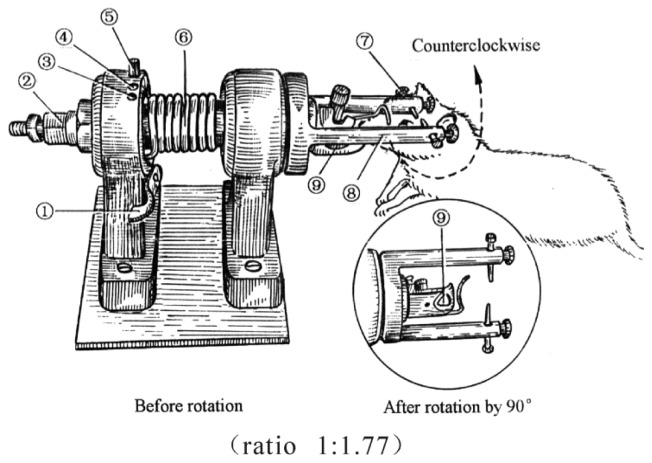
Diagram of the device to induce traumatic brain injury (TBI) by lateral head acceleration (ratio 1:1.77). (1) trigger; (2) reset nut; (3) rotational angle-determined hole at 0°; (4) rotational angle-determined hole at 30° and its bolt; (5) rotational angle-determined hole at 30°; (6) driving spring; (7) ear pin; (8) horizontal arm; (9) anterior tooth hole.

TBI was induced by a lateral head acceleration device (Figure 7). All animals were anesthetized by inhaling ethyl ether, then their head was horizontally secured to the rotation device by two lateral ear bars, a head clip and anterior teeth holes with its body 20° oblique to the top of the laboratory table. For the TBI group, immediately after the rat restored its struggle, the device trigger was pressed causing the rapid rotation of the rat head by 90° angle laterally (*i.e.*, in coronal plane). The head rotation finished in less than 2.09 ms, with an angular velocity of 761 rad/s and an angular acceleration of 1.87 × 10^5^ rad/s [27].

### 4.3. Immunohistochemical Staining

Both experimental and control rats were anesthetized with an intraperitoneal injection of pentobarbital sodium (50–60 mg/kg), and then exsanguinated by transcardial perfusion with 4 °C heparinized saline until the circulating effluent became transparent. The rats were fixed transcardially by 4% paraformaldehyde in PBS for 3 h, with perfusion pressure maintained at 90–95 mmHg and temperature at 37 °C. Next, each animal was decapitated and its brain was removed immediately and immersed in 4% paraformaldehyde overnight. After being washed many times, brain samples were routinely dehydrated and embedded in paraffin. Two micrometer thick sections were cut and dried at 8 °C for 1 h and kept at 70 °C overnight. Next, the sections were placed in boiling water at higher pressure for 2 min and 15 s. The sections were processed with SABC (StreptAvidin-Biotin Complex) and DAB (3,3′-diaminobenzidine tetrahydrochloride) color reagent kits from Zhongshan Agent Corporation, Beijing, China as per protocols. The tissues were probed with a mouse monoclonal primary antibody against S100A6 (1:50; Abcam, Suite B2304, Cambridge, MA, USA). The negative control was incubated in 0.01 mol/L PBS. The sections were observed by light microscope.

### 4.4. Western Blot

Rats were decapitated under pentobarbital anesthesia as previously described. Hippocampal tissues were removed and homogenized in sodium dodecyl sulfate (SDS) sample buffer (KeyGEN, Nanjing, China) containing a mixture of proteinase inhibitors. Protein samples were separated on SDS-polyacrylamide gel electrophoresis (SDS-PAGE) gels (4%–15% gradient gel; Bio-Rad, Hercules, CA, USA) and transferred from gels to nitrocellulose membranes. Blots were blocked for 1 h in a Tris-buffered saline solution with 0.1% Tween-20 (TBST) and 5% skim milk, and then incubated overnight at 4 °C with a primary antibody against S100A6 (1:1000; mouse monoclonal, Abcam, Suite B2304, Cambridge, MA, USA). Then the blots were incubated with an HRP (Peroxidase Horseradish)-conjugated secondary antibody (1:20,000; Beijing Zhongshan Biotechnology, Bejing, China) for 1 h at room temperature. Blots were visualized by incubation in enhanced chemiluminescence (ECL) Western blotting substrate solution (Pierce Biotechnology, Shanghai, China) for 1 min and followed by exposure to hyperfilm for 1–10 min. The blots were then incubated in a stripping buffer (67.5 mM Tris, pH 6.8, 2% SDS, and 0.7% β-mercaptoethanol) for 30 min at 50 °C and reprobed with a polyclonal mouse anti-β-actin antibody (1:1000; Abcam, Suite B2304, Cambridge, MA, USA) to serve as a control for sample loading. The Western blot analysis was conducted in triplicate. The density of specific bands was measured using an imaging analysis system (Fotodyne, Hartland, WI, USA) and normalized against corresponding loading controls. The ratio of S100A6 to β-actin in intensity (OD value) was regarded as the protein level for a tissue sample.

### 4.5. RT-PCR

Total RNA was isolated from 10^7^ unsorted or 2 × 10^5^ sorted thymocytes with Tri Reagent (Beijing Zhongshan Biotechnology, Beijing, China). To generate the most sensitive and biologically relevant results, sample collection, storage, and RNA isolation were performed according to the guidelines and instructions for maximizing the yield and quality of sample RNA. The final RNA precipitate was dissolved in diethylpyrocarbonate (DEPC)-treated water and the RNA content, and purity was checked by UV spectrophotometry at 260 and 280 nm. To eliminate DNA-contamination of the RNA, the samples were digested with DNase before reverse transcription. Reverse transcription was performed using 1 μg RNA/sample in the presence of 0.5 μg oligo (dT)16, M-MLV RT 1× reaction buffer (Promega Corporation, 2800 Woods Hollow Road, Madison, WI, USA), 1 mM of each dNTP, 40 units of RNase Inhibitor, and 1 unit of M-MLV reverse transcriptase (Promega Corporation, 2800 Woods Hollow Road, Madison, WI, USA), Negative controls included all of the aforementioned components except the reverse transcriptase enzyme. Primers were designed for S100A6 and GAPDH (S100A6 Forward 5′-CAGATGAGGGGAGACTCGTCA-3′; Reverse 5′-TGGCTTACACAACGCACATTC-3′ Length 154 GAPDH Forward 5′-GGCACAGTCAAGGCTGAGAATG-3′; Reverse 5′-ATGGTGGTGAAGACGCCAGTA-3′ Length 161). A Bio-Rad CFX96 Real-Time PCR machine set for 40 cycles amplified the RNA. After step 1: 95 °C for 30 s, steps two to four were repeated 40 times (step 2: 95 °C for 5 s, step 3: 60 °C for 30 s, step 4: A plate reading), before finishing with step 5: A melt curve 65 to 95 °C, incremental reduction in temperature by 0.5 °C for 5 s, and a plate reading. The relative normalized expression of S100A6 was recorded. Bio-Rad CFX Manager software (3.0 Bio-Rad Company, Hercules, CA, USA) was used to analyze the data of the real-time PCR and generate graphs.

### 4.6. MWM

MWM test involves forcing an animal to swim in order to find a platform hidden in the water. It measures their learning and memory capacity for spatial orientation [28]. The maze used in this study consisted of a black-painted circular pool (2.0 m in diameter, 0.5 m deep) filled with water. The water was maintained at a depth of 0.30 m and a temperature of 22 °C. The pool was surrounded by a curtain containing contrasting shapes as spatial cues. A black-colored circular platform, which was 9 cm in diameter and made of acrylic resin, enabled animals to escape from the water. It was submerged 1 cm under the water level and fixed in a constant position (*i.e.*, halfway between the center and the edge of the pool). The pool was divided into four quadrants, each of which was subdivided into two equal parts. The swim-route was recorded by a video camera placed 2 m above the center of the pool and connected to a video analysis system (DigBeh-MR, Shanghai Auspicious Software Technology Company Limited, Shanghai, China). For each trial, the rat was placed into the water at a half quadrant facing and close to the inside wall of the pool and was freed. The rat would swim to find a platform to escape the water. For each animal in a trial, the interval time between being freed in the water and climbing onto the escape platform was recorded as escape latency. Starting time and location for daily training was counter-balanced among rats and groups. Escape latency (the average of four trials per day) for TBI and sham groups over the following observational days were plotted to generate a learning curve. A MWM probe test was given 3 days after the 4-days MWM trial. The hidden platform was removed from the pool and each animal was allowed to swim for 60 s starting from the same position. “Platform crossing” referred to the times a rat swam over the position in which the platform had been originally located in its attempt to escape the water. Shapes on the curtain provided cues to assist rats’ search for the target. Platform crossing was used to assess the cued reference memory of both TBI- and sham-rats. Animal maze performance, including escape latency, platform crossing, and swim speed were analyzed via computer software. Animals were excluded from the experiment if they failed to swim and find the platform.

### 4.7. Statistical Analysis

Data were expressed as mean ± standard error of the mean (SEM), and analysis of variance (ANOVA) was calculated. Graphpad Prism software (v.5.01, Graphpad software, San Diego, CA, USA) was used to analyze the data and generate the graphs. TBI- and sham-rats’ performances were compared, as well as TBI subgroups. Significance level was set at *p* < 0.05.

## 5. Conclusions

In conclusion, using a rat model of TBI, our data from immunohistochemical staining, Western blot, and RT-PCR of S100A6 all indicates that the expression of S100A6 in hippocampus is reduced significantly at 1–6 h post-injury, and then gradually returns to baseline at 14 days. These protein and mRNA changes are accompanied by a relatively delayed deficiency in spatial learning and memory. Our data seems to suggest that down-regulation of S100A6 is involved in early posttraumatic events that lead to secondary cognitive disorders. The elevation of S100A6 levels is implicated in neuronal regeneration and repair in functions. S100A6 level alterations in rat hippocampus post-TBI occurred in association with animal cognitive deficits. The conclusions of this exploratory analysis are based, however, on limited experimental findings and lack supporting causal evidence. Also, the design of the study involving multiple testing could lead to statistical errors. Moreover, in our previous publication [27], no obvious morphological differences between left and right half brains were observed with the rat TBI model due to lateral head rotation. It was supposed that rat brains with a very small mass produced no left and right differences that were easily detectable under an extremely strong rational acceleration. Although we counterbalanced the left and right brain in both immunohistochemistry and Western blotting treatment in the present experimental design in order to reduce such a limitation caused by different brain laterality, a more strict protocol using either left or right half brains should have been taken in tissue preparation and comparison. Hence, the present report should be considered only a preliminary investigation that provides valuable clues to hopefully give rise to further, well-controlled studies. Further exploration is needed to investigate the underlying molecular mechanisms involving S100A6 in the initiation of the cascade of events immediately following TBI.
